# Food Taboo and associated factors among pregnant women attending antenatal clinics at Bahir Dar City, North West Ethiopia, 2021: cross-sectional study

**DOI:** 10.1038/s41598-023-34964-5

**Published:** 2023-05-13

**Authors:** Meseret Abere, Abebaw Gedef Azene

**Affiliations:** 1 Department of Nutrition, Bahir Dar Institution of Technology, Bahir Dar University, Bahir Dar, Ethiopia; 2 Department of Epidemiology and Biostatistics, School of Public Health, College of Medicine and Health Science, Bahir Dar University, Bahir Dar, Ethiopia

**Keywords:** Health care, Risk factors

## Abstract

Food taboo is any unacceptable food items in the society that arise mainly based on religious, cultural, historical and social principles. Developing countries faced the triple burden of malnutrition of under nutrition, micronutrient deficiencies and overeating. Food taboos have great effect on pregnant women through prohibited essential food and/or drinks. There is a paucity of study conducted in food taboo practice among pregnant women in Ethiopia. This study aimed to assess the prevalence of food taboo practice and associated factors among pregnant women attending antenatal care (ANC) at Bahir Dar city, 2020. Institutional based cross-sectional study design was conducted among 421 pregnant women attending antenatal care clinics. Stratified sampling technique was used to approach the study participants, and interviewer administered questionnaire was used for data collection. Binary logistic regression analysis was conducted to identify predictors. The prevalence of food taboo practices among pregnant women was 27.5% (95% CI 23.2–31.8%) at the Bahir Dar city. Most food items avoided during pregnancy were meat, honey, milk, fruit and cereals. Reasons for avoidance of these food items were plastered on the fetal head, and making fatty baby which is difficult for deliver. Maternal age 20–30 years (AOR = 8.39, 95% CI 3.49–20.14), more than 30 years [AOR = 10.56, 95% CI (2.00, 51.74)], more than 2 parity [AOR = 9.83 95% CI (2.79, 34.70)], no previous experience of the ANC visit [AOR = 2.68, 95% CI (1.26, 5.73)], and no information about nutrition [AOR = 4.55, 95% CI (1.77, 11.70)] were significantly associated with practice of food taboo. This study revealed that prevalence of food taboo is high during pregnancy. The implications of this study that needs strengthening nutrition counseling components of ANC follow-up and health professionals needs to design and implement strategic health communication intended to reorient misconceptions and myths for the pregnant women regarding the food taboo.

## Introduction

Maternal nutrition has a huge impact on the health of the mother and the fetus. During pregnancy all women need more food, a varied diet, and micronutrient supplements to get additional 300 extra calories per day for baby’s growth and development^1^. Energy needs increase from first to third trimester of pregnancy. Inadequate weight gain during pregnancy often results in low birth weight, which increases an infant’s risk of dying^2^. Pregnant women health depends greatly on the availability of food and, who have poor nutritional status are at higher risk of infectious disease such as malaria, and infestation with gastro intestinal parasites and death^3^.

Food taboo is a prohibition against consuming certain foods. The word "taboo" is *Polynesian* and means sacred or forbidden; it has a quasi-magical or religious overtone^4^. It is unwritten social rules that regulate human behavior^5^. Food taboo is any consideration of food items by the society as improper or unacceptable that arises mainly based on religious, cultural, historical and social principles^2^.

Religions have a powerful influence on food taboos and declare certain food items fit and others unfit for human consumption. Some foods may be prohibited during certain religious periods at certain stages of life or to certain classes of people, even though the food is otherwise permitted^1^. Dietary rules and regulations govern particular phases of the human life cycle and associated with special events such as a menstrual period, pregnancy, childbirth, lactation, and in traditional societies preparation for the hunt, battle, wedding and funeral^6^.

Studies reported that food taboo accounts largely to maternal and fetal malnutrition during pregnancy^1,7–9^. Food taboo often applies to women and relate to the reproduction cycle^5^. Pregnant women are prevented from accessing a well-balanced diet, resulting in a high prevalence of low birth weight and harm to mother and baby health^6^.

Findings showed that nutrition during pregnancy was the single most important factor predicting maternal anemia, low body weight, iron deficiency, preterm birth, intrauterine growth restriction and reproductive loss through still births^10^. It is influenced by different factors like dietary counseling, whether attending antenatal care (ANC) clinic or not, younger age, less educational status, and multiparous and pregnant women^6,11–14^. Culture and belief also influence maternal eating patterns during pregnancy^2,15^.

Maternal malnutrition has been strongly linked to functional consequences like increased risk of adverse pregnancy outcomes, poor infant survival and risk of chronic diseases inlater stages of life^10^. To what extent such food taboos, associated factors, and misconceptions exist and how they affect pregnancy outcomes in Ethiopia is unknown. This study aimed to assess the prevalence of food taboo practice and associated factors among pregnant women attending antenatal clinics at a public health facility in Bahir Dar city. So this study provided relevant information about food taboo practice among pregnant women in the study area.

## Methods and materials

### Study area and period

Institutional based cross-sectional study design was conducted in Bahir Dar city at public health facilities from October 20-November 20/ 2020. Bahir Dar is the capital city of Amhara region in North West Ethiopia, located 565 km away from Addis Ababa, the capital city of Ethiopia. The city is situated with an elevation of 1,800 m (5,900 ft.). The average temperature and humidity of at Bahir Dar city is 26 °C and 31%, respectively, and the direction of wind is North West at 10 km. According to the report of the Bahir Dar city municipality, the total population of the Bahir Dar city in 2013 was 445,084. Of this population, 222,987(50.1%) were females^13^. There are three public hospitals and 10 health centers, all are providing ANC service for community.

### Source and study population

All pregnant women who were attending antenatal clinics at public health facilities in Bahir Dar city were the source population. All pregnant women who were attending ANC clinics at the selected public health facilities during the data collection period at Bahir Dar city from October 20-November 20/ 2020 were studied population.

### Inclusion and exclusion criteria

A pregnant woman who was attending ANC service in the selected facilities during the study period was included. The pregnant woman attending ANC in the selected facilities but lived out of the Bahir Dar city during the study period was excluded.

### Sample size determination and

The minimum sample size (proportion of women’s having a practice of food taboo) was calculated using single population proportion formula based on the following assumption: confidence level 95% ((Z = 1.96), proportion food taboo among pregnant women attending in Wondo Genet town was 49.8%^16^ and margin of error 5% was 383. Adding 10% non-response rate, the final sample size was 421 individuals.

### Sampling technique

Stratified sampling technique was used to approach the study participants in health centers at the study area. Then all pregnant women who visit the health facilities were recorded as a sampling frame in the selected health centers. Of the total 10 health centers and 3 hospitals, firstly, we stratified in two strata like health center and hospitals. Three health centers (Bahir Dar, Abay Mado and zenzelema health centers) and one hospital (Felege Hiwot specialized comprehensive hospital (FHSCH)) selected randomly. The calculated sample size was proportionally allocated to each selected health facility. Participants were selected randomly in each selected health center and hospital.

### Variables of the study

Practice of food taboo (Yes, No).

#### Socio demographic factors

Maternal age, family size, educational status, income, occupation, ethnicity, religion, marital status, and residence.

#### Reproductive characteristics

Parity and gravidity.

#### Need factors

Means of awareness of pregnancy, perception on timely booking of ANC, advice from significant others, type of pregnancy/wanted or unwanted.

### Operational definition

#### Time of ANC attendance

The first time by which pregnant mothers come to antenatal clinic to get care from health professionals.

#### Timely ANC initiation

The first ANC visit before 16 weeks of gestational age.

#### Late ANC initiation

First ANC visit start at or after 16 weeks of gestational age.

#### Food taboo

At least one food items averted during pregnancy.

### Data collection tools and methods

Structured and pretested interviewer administered questionnaire was used to collect the data from pregnant women who attending ANC. The questionnaire was developed in English then translated into local language Amharic. Six trained nurses and midwives and two BSc midwives were recruited as data collectors and supervisors for data collection process, respectively.

### Data quality assurance

Quality of data assured by using a properly designed questionnaire from literatures developed for similar purpose. The questionnaire was pre-tested on 5% of sample size prior to actual data collection at Merawi primary hospital. One day training was given for data collectors and supervisors on the rationale of the study, data collection technique and how to taking consent from respondents. Each questionnaire was reviewed daily by the supervisors and the principal investigator to check the completeness and clarity of the questionnaire immediately after received from the participants in the field.

### Data management and analysis

The collected data entered into Epi-Info version 7, and then exported to SPSS version 23 for cleaning and analysis. Descriptive statistics were computed and presented using frequency table, proportion, graph and tables. The association between independent and outcome variable was assessed using a binary logistic regression model. All explanatory variables with *P* < 0.20 in simple binary logistic regression analysis was candidate to multiple binary logistic regression analysis and significant association was declared based on *P* < 0.05 and odds ratio with 95% CI. No or little multicollinearity assumption was checked using variance inflation factor (VIF) less than 10. The overall model goodness of fit was assessed using a Hosmeur and Lemshow test (*P*_value = 0.62).

### Ethical consideration

Ethical clearance was obtained from the Institutional Review Board of Bahir Dar University, Ethiopia. Then legal official clearance letter was obtained from the Amhara public health institute (APHI). Finally, a legal official letter received from Bahir Dar zonal health department and each health facility. The study was conducted in strict accordance with the ethical standards set forth in the 1964 Declaration of Helsinki and the ethical review board of Bahir Dar University, Ethiopia. Informed written consent also obtained from each participant. Participants were informed about the purpose of study, the right refuses, or partial refuse. Confidentiality secured by avoiding writing the participant’s name and the data cannot accessible by a third person.

## Result

### Socio-demographic characteristics of the participants

In this study; a total of 421 pregnant women were participated with a 100% response rate. Of this, 25(5.9%) were less than 20 years age, 262(62.2%) were in the age group of 21- 29 years. One hundred eighty one (43%) participants were tertiary level and above followed by secondary education level (122(29%)). Moreover, regarding to their husband education, 206(48.9%) of the respondents had tertiary level and above, and 134(31.8%) had secondary education. Out of the respondents, 329(78%) were followers of Orthodox Christianity religion followed by Muslim who accounts since 70 (16.6%).

With regard to their ethnicity, 390(96.2%) were Amhara, 18(4.3%) were Oromo and 13(3%) were Guragie. House wife was the leading occupations (132(31.4%)) followed by civil servant 111(26.4%). Pertaining to the income distribution of respondents; 55(18.8%) had incomes less than Birr 2000, 65(22.2%) had incomes between Birr 2000–3500, 62(21.2%) had incomes between Birr 3501–5000 and 111 (37.9%) had incomes greater than 5000 (Table 1).

**Table 1:** Socio Demographic characteristics of pregnant women in Bahir dar city, Ethiopia 2020 (n = 421).

Socio demographic characteristics	No	%
Educational status		
Unable to read and write	64	15.2
Read and write	54	12.8
Secondary	122	29
Tertiary and above	181	43
Educational status of husband		
Unable to read and write	36	8.6
Read and write	45	10.7
Secondary	134	31.8
Tertiary and above	206	48.9
Resident		
Rural	73	17.3
Urban	348	82.7
Marital status		
Married	405	96.2
Divorced	16	3.8
Religion		
Orthodox	329	78.2
Muslim	70	16.6
Protestant	22	5.2
Ethnicity		
Amhara	390	92.6
Oromo	18	4.3
Guragie	13	3.1
Occupation		
Unemployed	57	13.5
Student	21	5
House wife	132	31.4
Daily laborer	25	5.9
Business man	45	10.7
Civil servant	111	26.4
Private work	8	1.9
Others	22	5.2
Age group		
≤ 20	25	5.9
21–29	262	62.2
≥ 30	134	31.8
Family size group		
≤ 3	227	54.4
> 3	190	45.6
Income Group in Ethiopian Birr		
≤ 2000	55	18.8
2000–3500	65	22.2
3501–5000	62	21.2
> 5000	111	37.9

Secondary: grade 9 to 10, tertiary and above: grade 11 and above, Business Man: dealer.

### Reproductive and nutrition characteristics

Number of pregnancies so far (gravidity), 160(38%) of the respondents had gravidity for two times and 107(25.4%) of the respondents had gravidity for a one time. Moreover, regarding to the status of parity, 155(37.2%) of the respondents had parity for one time 93(22.3%) of the respondents had parity for two times and 133(31.9%) of the respondents had no experienced of parity.

In addition, 78(19.8%) of the respondents have experienced abortion. Among pregnant women 232(80%) of the pregnant women visited ANC previously. Out of 232 respondents, 128(67%) were visited four times and above, 121(52.2%) of the women started ANC at 1^st^ trimester, and 357(85.8%) of the women having nutrition information about the importance of dietary diversity during pregnancy.

More than half (58.4%) of pregnant women practice of fasting during pregnancy, type of fasting includes restriction of meat and milk containing food items in orthodox religion and abstain from eating at daytime in Muslim women (Table 2).

**Table 2:** Reproductive and nutrition characteristics of pregnant women in Bahir dar city, Ethiopia 2020 (n = 421).

Variables	No	%
Gravidity		
0	10	2.4
1	107	25.4
2	160	38
3	96	22.8
≥ 4	48	111.4
Parity		
0	133	31.9
1	155	37.2
2	93	22.3
3	36	8.6
Abort		
Yes	78	19.8
No	315	80.2
ANC visited in the previous pregnancy		
Yes	232	80
No	58	20
Number of ANC Visited base on trimester		
1st visit	8	4.2
2nd visit	5	2.6
3rd visit	51	26.6
4th and above	128	67
Time of ANC started		
1st Trimester	121	52.2
2nd Trimester	98	42.2
3rd Trimester	13	5.6
Do you have an information about dietary diversity is important during pregnancy		
Yes	357	85.8
No	59	14.2
Fasting during pregnancy		
Yes	240	58.4
No	171	41.6

### Prevalence of food taboo among pregnant women

In this study; we found that the prevalence of food taboos among pregnant women was 112 (27.5% (95% CI 23.2–31.8%)). Furthermore; we found that the types of food desisted from by pregnant women were 80 (22.5%) avoided eating meat, 76(21.4%) of them avoided eating eggs, 59(16.6%) avoided eating honey, 36(10.1%) avoided drinking milk, 60(16.9%) of them avoided eating fruit, and 44(12.4%) of them avoided eating cereals. Even though those food types are so essential for pregnant women, they practiced abstain from those foods during pregnancy. Out of those who abstain food during pregnancy, they reason out that 48 (21.8%) their delivery was difficult and 58 (26.4%) of their baby were fatty, obesity, 65(29.5%) were plastered (Table 3).

**Table 3:** Items of Food taboo during pregnant women in Bahir Dar city, Ethiopia 2020.

Variables	No	%
Milk avoided	36	10.1
Egg avoided	76	21.4
Meat avoided	80	22.5
Honey avoided	59	16.6
Cereal avoided	44	12.4
Fruit avoided	60	16.9
Plastered	65	29.5
Fear of fatty	58	26.4
Delivery problem	48	21.8

### Factors associated with practices of food taboo

We assessed the association between each independent variable with practice of food taboo during pregnancy. The variables such as, age group, family size, educational status, gravidity, parity, ANC visit, information about dietary diversity is important during pregnancy and practices of fasting during pregnancy were associated with the dependent variable but monthly income of pregnant women, status of husband education, abortion, and time of ANC started failed to maintain their association with the dependent variable in the binary logistics regression.

After adjusting for the effect of confounding variables using multiple binary logistics regression analysis, variables like age of pregnant women, parity, ANC visit during last pregnancy, and information about dietary diversity during pregnancy were statistically significant association with the practices of food taboo while the rest variables were not statistically significant at p-value < 0.05.

In the multivariate logistic regression analysis as the age of the woman is increased, adoption of the food taboo is increased. Pregnant women whose age is 20–30 years were 8.39 times more likely to develop food taboos compared with the age less than 20 years (AOR = 8.39, 95% CI 3.49–20.14). And also pregnant women whose age was more than 30 years had 10.56 times more likely practices of food taboos as compared to those age group less than 20 years [AOR = 10.56, 95% CI (2.00, 51.74)]. Pregnant women, those who had more than 2 parity were 9.83 times more likely practices of food taboos as compared to than those who had less than 2 parity [AOR = 9.83 95% CI (2.79, 34.70)].

Moreover, the pregnant women who had no previous experience of ANC visit were 2.68 times more likely developed food taboos as compared to those who had an ANC visit during last pregnancy [AOR = 2.68, 95% CI (1.26, 5.73)]. Pregnant women who had no information about nutrition during pregnancy were 4.55 times more likely developed food taboos as compared to those who had information about nutrition [AOR = 4.55, 95% CI (1.77, 11.70)]. (Table 4).

**Table 4:** Binary and multivariate logistic regression analysis of factors associated with practices of food taboos in Bahir Dar city, Ethiopia, 2021.

Variables	Food Taboo Practice							
	Yes	No	COR(95% C.I)			AOR(95% C.I)		
Age group								
< 20	5	20	1			1		
20–30	60	192	2.27	0.80	6.43	8.39	3.49	20.14
> 30	47	83	1.81	1.14	2.87	10.16	2.00	51.74
Family size group								
1–3	74	143	2.02	1.28	3.17	1.66	0.63	4.40
> 3	38	148	1			1		
Educational status								
Illiterate	25	35	1.94	1.05	3.59	1.20	0.24	2.90
Read and Write	14	40	0.95	0.47	1.91	0.00	0.00	
Secondary	27	95	0.77	0.45	1.33	0.45	0.17	1.21
Tertiary	46	125	1			1		
Gravidity								
≤ 2	83	183	1.75	1.08	2.84	2.65	0.78	8.99
> 2	29	112	1			1		
Party								
≤ 2	98	273	1			1		
> 2	14	18	2.17	1.04	4.52	9.83	2.79	34.70
ANC visit								
Yes	56	176	1			1.00		
No	23	31	2.33	1.26	4.32	2.68	1.26	5.73
Food information								
Yes	88	264	1			1		
No	24	31	2.32	1.29	4.17	4.55	1.77	11.70
Fasting								
Yes	48	183	1			1.69	0.87	3.28
No	64	107	2.28	1.46	3.55			

## Discussion

This study aimed to assess the practice of food taboo and associated factors among pregnant women attending antenatal clinics at a public health facility in Bahir Dar City, Northwest Ethiopia, 2020.

This study found that the prevalence of food taboo was 27.5%. This finding is similar to the study conducted in the Awabel District in West Gojjam, which was 27%^17^. This finding is much lower than studies conducted at Shashemene and Wodogenet in Ethiopia which were 49.8% and 44.8%, respectively^1,17^. This prevalence also much lower as compared to in North Costal Paradesh, it was 82.1%^19^. The possible reason for this difference is may be related to the awareness and knowledge of mothers increase due to time and education level. Another reason perhaps the difference of culture, religion and socio economic background of study participants of the studies area^17^. Moreover, the prevalence of food taboo observed in this study is therefore relatively greater than when as compared to report elsewhere in Ghana, Accra, Africa^18^. It is thought that the relatively low prevalence of food taboos observed in Ghana is due to cultural influence or and religious impact.

Regarding to the type of food items which were avoided in the meal were milk, egg, meat, honey, cereal and fruit. Even though those food types are so essential for pregnant women, out of those who abstain food during pregnancy, they reason out that 48 (21.8%) their delivery was difficult and 58 (26.4%) of their baby were fatty (Obesity), and 65(29.5%) were plastered. This result is consistent with the study conducted in Awable District, East Gojjam^17^ and Addis Ababa^19^. Practically, 112 women avoided livestock products such as meat and milk, this is one of the serious disadvantages of observing food taboos since the major sources of protein which are essential nutrients needed for the rapidly growing fetus are avoided. The study also showed that these women did not take adequate egg, fruit and cereals. The magnitude of the high intake of egg, fruit, and vegetable in this study was small. As researchers suggest that a dietary pattern characterized by high intake of vegetables, plant foods, and vegetable oils decreases the risk of preeclampsia^20^.

Furthermore; we assessed the association between the practices of food taboos and the independent variables: age, family size, gravidity, parity, abortion, ANC visit, information about nutrition and practices of fasting.

In this study; we found that the age of the mother was significantly associated with the practices of food taboos. As age of the mother increases, practices of the food taboo also increase. Pregnant women whose age were 20–30 years were 8.39 times more likely to develop food taboos compared with the age less than 20 years (AOR = 8.39, 95% CI 3.49–20.14). And also pregnant women whose age was more than 30 years had 10.56 times as likely to practice food taboos as compared to those age group less than 20 years [AOR = 10.56, 95% CI (2.00, 51.74)]. This finding is consistent with other studies conducted in Awabel, and Shashemenie which stated that women more than 35 years age were more likely to practice food taboos^1,17^. The possible explanation could be younger women may be more likely to accept modern health services since they are more energetic and more likely to attend formal education. Older women on the other hand, tend to believe on indigenous knowledge of traditional practice thus giving less attention to eat balanced diet^21^.

Similarly; pregnant women, those who had less than or equal to 2 parity were 9.83 times as likely to practices of food taboos as compared to those who had more than 2 parity [AOR = 9.83., 95% CI (2.79, 34.70)]. The study, conducted in Awabel district, Gojjam, Ethiopia, also showed that statistically significant association between parity and practices of food taboos during pregnancy^17^. But this finding is contradict with studies conducted in South Africa, and Eastern Nigeria, the possible reason for this difference may due to countries cultural and socio-economic difference^5,11,16,22^.

The finding of this study also revealed that previous ANC attendance was significantly associated with practice of food taboo. Pregnant women who had no previous experience of ANC visit were 2.68 times as likely to practice food taboo as compared to those who had an ANC visit during last pregnancy [AOR = 2.68, 95% CI (1.26, 5.73)]. This result is in line with the study conducted in Awebel^17^, which found that pregnant women who have never had ANC attendance in the health institution were 2.33 times more likely to develop food taboo as compared with those who have had ANC attendance. This may be due to the knowledge gained from formal education and experienced health education. Moreover; pregnant women who had no information about nutrition during pregnancy were 4.55 times more likely to develop food taboos as compared to those who had information about nutrition [AOR = 4.55, 95% CI (1.77, 11.70)]. This result supported by the fact that good knowledge about basic nutrients and adequate well balanced diet usually resulting in positive dietary practices which are important determinants of optimum health pregnant women Shashemene^1,17^, Awabel^17^ and rural Central Ethiopia^13^.

## Conclusion

This study revealed that prevalence of food taboo is high during pregnancy. Age of the mother, parity, previous ANC attendance, available of information had significant association with food taboo. The implications of this study that needs strengthening the nutrition counseling components of ANC follow-up and health professionals needs to design and implement strategic health communication intended to reorient misconceptions and myths for the pregnant women regarding the food taboo.

## Data Availability

All data are available from the first author upon reasonable request.

## Abbreviations

ANC: Antenatal Care
APH: Amhara Public health
CI: Confidence Interval
DDS: Diversity Diversification Score
FHSCH: Felege Hiwot specialized comprehensive hospital
LBW: Low Birth weight
